# Estimating receptive fields and spike-processing neural circuits in Drosophila

**DOI:** 10.1186/1471-2202-13-S1-O10

**Published:** 2012-07-16

**Authors:** Aurel A Lazar, Yevgeniy B Slutskiy

**Affiliations:** 1 Department of Electrical Engineering, Columbia University, New York, NY 10025, USA

## 

One of the long-term goals of sensory neuroscience is the development of sound experimental and theoretical methods for understanding the functional organization of sensory systems. In this regard, *Drosophila melanogaster* is the model organism of choice: it boasts a relatively small brain, its sensory systems have been anatomically well characterized and it offers an extensive genetic toolbox for visualizing and altering its neural circuits. Despite these advantages however, comprehensive models of sensory processing in *Drosophila* are sparse, in particular due to the lack of methods for estimating spike-processing neural circuits in higher brain centers. The majority of existing neural circuit models and methods for their identification assume rate-based systems (see [1] for a review), and take both the input (stimuli) and the output (response rates) to be in the continuous domain. In a practical setting, however, outputs of most neurons in a sensory system are sequences of all-or-none action potentials. Furthermore, input signals are continuous only for those neurons that are located at the sensory periphery. In contrast, input signals for neurons upstream of sensory neurons are spatiotemporal spike trains. Hence, there is a need to develop a generic framework for estimation of both receptive fields in the periphery and of spatiotemporal spike processing upstream.

Here we propose a novel theoretical approach for estimating receptive fields in circuit models that incorporate biophysical spike-generating mechanisms (e.g., the Hodgkin-Huxley neuron) and admit both continuous sensory signals and multidimensional spike trains as input stimuli. We thus explicitly take into account the highly nonlinear nature of spike generation that has been shown to result in significant interactions between various stimulus features [2], [3] and to fundamentally affect the estimation of receptive fields [4]. Furthermore, and in contrast to many existing methods [1], our approach estimates receptive fields directly from spike times produced by a neuron, thereby obviating the need to repeat experiments in order to compute the neuron’s instantaneous rate of response (e.g., PSTH). The employed test signals belong to spaces of bandlimited functions and bridge the gap between identification using synthetic and naturalistic stimuli. This makes our methodology particularly attractive in those sensory modalities (most notably olfaction [5]), where it is difficult to produce stimuli that are white and/or have particular distribution/ attributes [1]-[4]. First, we work out in detail algorithms for identifying temporal, spatial and spatiotemporal receptive fields in the sensory periphery. We show that our methodology is readily generalizable to multiple receptive fields as well as to higher dimensions, allowing one to consider more complex receptive fields, if needed. Second, we demonstrate how to identify the processing of multiple spiking inputs converging onto the dendritic tree of a spiking neuron. Third, we show that the presented methodology allows one to model integration of sensory modalities in higher brain centers. Finally, we test the proposed approach using *in-vivo* data recorded from the olfactory system of *Drosophila*.
